# A New Setup for Real-Time Investigations of Optical Fiber Sensors Subjected to Gamma-Rays: Case Study on Long Period Gratings

**DOI:** 10.3390/s20154129

**Published:** 2020-07-24

**Authors:** Andrei Stancalie, Flavio Esposito, Constantin Daniel Neguț, Marian Ghena, Razvan Mihalcea, Anubhav Srivastava, Stefania Campopiano, Agostino Iadicicco

**Affiliations:** 1 National Institute Laser, Plasma and Radiation Physics, Center for Advanced Laser Technologies (CETAL), 409 Atomiştilor St., RO-077125 Măgurele, Romania; andrei.stancalie@inflpr.ro (A.S.); marian.ghena@inflpr.ro (M.G.); razvan.mihalcea@inflpr.ro (R.M.); 2 Department of Engineering, University of Naples “Parthenope”, Centro Direzionale Isola C4, 80143 Naples, Italy; anubhav.jackie@uniparthenope.it (A.S.); stefania.campopiano@uniparthenope.it (S.C.); iadicicco@uniparthenope.it (A.I.); 3 “Horia Hulubei” National Institute for R&D in Physics and Nuclear Engineering, 30 Reactorului St., RO-077125 Magurele, Romania; dnegut@nipne.ro

**Keywords:** gamma radiation, long period gratings, optical fiber sensors, dosimetry

## Abstract

In this work, we present a new setup for real-time investigations of optical fibers and optical fiber sensors while being subjected to gamma-rays. The investigation of the radiation effects on novel or well-assessed sensing devices has attracted a lot of interest, however, the facilities required to do this (when available) are barely accessible to the device to be characterized. In order to reduce the limitations of these types of experiments and ensure a highly controlled environment, we implemented a configuration that permits the on-line testing of optical components inside a Co-60 gamma chamber research irradiator. To show the advantages of this new approach, we present a case study that compares an arc-induced optical fiber long period grating (LPG) irradiated in a gamma chamber with the same type of grating irradiated with gamma-rays from a Co-60 industrial irradiator. In order to better understand the effects of radiation on such components and their behavior in radiation environments, we focus on the homogeneity of the radiation field and parameter customizability as well as the high reproducibility of the experiments.

## 1. Introduction

Fiber optic sensing technology has been extensively studied due to its vital role and potential in industrial applications. Optical fiber sensors are typically sensitive to temperature [1], strain [2], bending [3] and the surrounding medium, thus, the ability to integrate them in harsh environments has been highlighted by the R&D sector, and in some cases, exploited by industry [4,5]. Due to the flexibility of optical fiber manufacturing technologies [6], and because optical fiber sensors can withstand a high level of radiation [7], there is great interest in their application in such environments [8,9]. Fields such as the space industry, high-energy physics, nuclear industry and related facilities have benefited from the advantages and flexibility of fiber-based technologies and have implemented such sensing solutions. 

Radiation-induced absorption (RIA) [10] and refractive index change [11] are the most investigated radiation-induced effects on optical fibers, depending on the working principle of the sensor. These effects are complex and are dependent on the chemical composition of the optical fibers, the environment in which the irradiation takes place and the manufacturing process [12]. Thus, optical fiber sensors that operate in the ionizing radiation field [13] have been extensively studied in order to define and understand the effects of radiation on their properties [14,15]. Investigations have focused on off-line and real-time monitoring, to assess ionizing radiation sensitivity of in-fiber devices and to correlate their response with the radiation dose [16,17]. Attention has also been paid to the radiation effects on standard optical fibers as compared to those with special compositions [18,19]. Among the different kinds of sensors, fiber gratings have attracted a lot of interest, and previously published works have demonstrated that exposure to gamma radiation will typically increase the refractive index in the fiber core (due to the presence of dopants) [20]. Depending on the composition of the fiber, this may limit the fiber selection which is based on the materials employed for core fabrication and the photosensitivity of dopants [16,21].

Various experimental setups have been implemented for the real-time monitoring of optical fiber induced attenuation and optical fiber sensor response, as a consequence of gamma irradiation. The on-line recording of the results allows a proper evaluation of the temporary effects as well as insights into the cumulative effects [22,23]. For example, the evaluation of combined radiation and temperature effects on single-mode Ge-doped and F-doped optical fibers was performed at the IRMA 60-Co facility, Institute de Radioprotection et de Sûreté Nucléaire (France) [14]. The samples were placed in a circle around the Co-60 rod to obtain an equal distribution of both temperature and radiation. Samples were subjected to gamma-rays with an average dose rate of 3.3 kGy/h depending on the position, while the temperature, monitored with three thermocouples, was set from room temperature up to 120 °C. Special polyamide coatings were utilized to increase the temperature tolerance of the devices. The investigation of RIA in single mode F-doped radiation hardened fibers reported by Y. Kim et al. [24] was based on a different type of 60-Co radiation source (MSD Nordion, pencil type/C-188 sealed). In this case, the optical fibers were placed parallel to the source while Pb plates were utilized to shield the connection for gamma exposure and consequent optical losses. The irradiation was performed 14 times and intermittently, while the equipment (ASE source and optical spectrum analyzer) was placed outside the irradiation zone. The reported dose value was approximatively 10 kGy for around one hour of irradiation. A different gamma irradiator type was reported in [25] where two pure silica cores with F-doped cladding polarization maintaining (PM) fibers were subjected to gamma radiation up to a dose of 1 kGy and a dose rate between 0.87 and 1.0 Gy/s depending on the position of the fibers. RIA evolution was recorded in real-time while post-irradiation recovery tests were carried out as well. The authors found that there was a significantly higher RIA in PM compared to reference non-PM fibers. The devices under test were placed in front of cobalt rods that were lifted from the ground (shielded zone) during irradiation. Optical interrogation equipment consisting of a halogen lamp and diode array spectrometers was placed and connected with peripherals, behind a 2 m thick concrete wall. Another application [21] focused on the real-time evaluation of the neutron flux and the gamma-ray mixing effect on the spectral response and sensitivity of several arc-induced long period gratings (LPGs). The experimental setup was developed around a “TRIGA” pulsed nuclear reactor, where the fibers were subjected to gamma radiation with dose rates of 9 Gy/s and an average neutron flux of 1.2 × 10^12^ n/(cm^2^s) for about two hours, resulting in a total dose of about 65 kGy and a neutron fluence of 9.18 × 10^15^ n/cm^2^. Many of the difficulties that have arisen in previous studies are strongly related to placing the setup near the radiation source, which creates the risk of damaging the sensors or inducing strain related errors.

In the abovementioned examples, the accuracy of the results is dependent on the capacity of setup to physically separate the external factors such as temperature or strain-induced variations. In almost all cases, the results obtained for optical fiber sensors are cumulative effects; thus, corrections are applied after the experimental data is acquired [26,27]. When subjecting fiber sensors to ionizing radiation inside industrial facilities [16], several factors need to be kept under observation and considered for correction as follows. Temperature variations may prove to be one of the most important secondary effects when it comes to the real-time investigation of optical fiber grating sensors subjected to radiation [28]. By performing laboratory calibration prior to irradiation, red- or blue-shifts in grating resonance wavelengths may be found because this parameter might be dominant compared to the radiation induced effects. For example, when considering a Co-60 source stored in a water pool, as in the case of an industrial irradiator, temperature fluctuations of 1–2 °C may occur when the source is lifted outside the water tank due to the difference between the water and room temperature. Moreover, in the same situation, the dose rate is dependent on the product being processed and can vary by 20%, mainly due to the product density and the loading pattern in the irradiation container. Strain variations are likely to be induced in a setup deployed inside a nuclear reactor as the components must be mechanically introduced at a distance quite far from the interrogation equipment. The channel that permits the setup to be placed near the reactor core has different geometries, and often S-shaped serpentines instead of straight channels are used to provide a barrier for radiation. Special care should also be directed to the optical connections and peripherals. Protection from radiation induced effects on standard connecting optical fibers is essential, as well as the protection of splices or adaptor parts of the setup. Additionally, factors such as changes in relative humidity must be considered especially with regard to plastic optical fibers [29] as well as the homogeneity of radiation field. These are key elements in experiments that are carried out in harsh environments where the position of the probes is not arbitrary or objects are interfering in the radiation path [30,31].

In this study, we design a new experimental setup for gamma irradiation of optical components based on optical fibers. Compared to previous configurations, the advantages of the new setup are the dose rate stability, thermal stability (the thermal regime), and constant and quantifiable optical connections, which make it possible to easily reproduce the experimental conditions. The stability of the parameters allows a more accurate study of optical fibers and gratings under gamma radiation [32], and also opens the possibility of calibrating these components in harsh environments [33]. The optical monitoring of the components is performed in real-time, from a close but safe distance via connecting fibers. The accumulated dose is validated with extreme accuracy using alanine dosimeters placed near the samples. The temperature in the proximity of the sample is well controlled and the thermal profile is available in real-time. Another advantage is that exposure is constant and totally customizable by the operator, without disturbing factors from outside the exposure zone. The presented method should prove to be extremely useful in research studies where: (a) dose rate needs to be customized; (b) exposure time needs to be precise; and (c) post-irradiation conditions need to remain stable (e.g., temperature, humidity, strain). To better demonstrate advantages of the proposed approach, we present a comparative experimental study on gamma irradiation effects on long period fiber gratings fabricated by the electric arc discharge (EAD) technique in standard SMF28 optical fiber [34,35].

## 2. Experimental Setup and Methods

### 2.1. Irradiation and Measurement Setups

The irradiation was carried out at the “Horia Hulubei” National Institute of Nuclear Physics and Engineering, Magurele, Romania using two types of gamma irradiators. Results obtained from the real-time monitoring of the radiation effects of a GC-5000 Co-60 (B.R.I.T., Maharashtra, India) research gamma irradiator were compared to the results obtained by using an industrial SVST Co-60/B gamma irradiator (Institute of Isotopes, Budapest, Hungary). An Alanine-EPR dosimetry system was utilized for the research irradiator by placing dose monitors near the samples under test. In the case of the industrial irradiator, interruptions of irradiation are frequent due to the exchange of products to be irradiated and the dose rates are ten times lower than those available in the research irradiator, thus, we used very stable chemical ethanol-chlorobenzene (ECB) dosimeters. Both dosimetry systems are traceable at NPL (Teddington, UK) via H.D.R.L (Roskilde, Denmark). 

**Figure 1:**
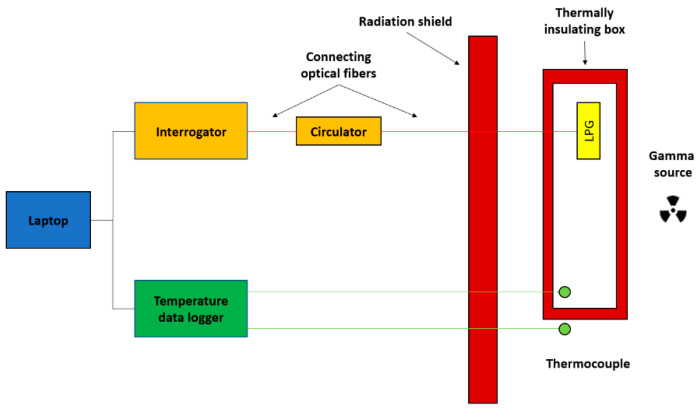
Irradiation setup utilizing the SVST Co-60/B irradiator. The irradiation zone and interrogation setup are separated by a concrete radiation shield. Long period gratings (LPGs) are placed in a thermally insulated box in the proximity of the Co-60 source, with thermocouple reference sensors.

The first irradiation setup (SVST Co-60/B), which was located in an industrial facility, is composed of a Co-60 gamma source stored in a water pool and it has been described in detail in our earlier works [16,36]. A sketch of this irradiation and measurement setup is detailed in Figure 1. Briefly, it is worth mentioning that the path from the optical interrogation units and the components involves multiple connections and is around 20 m in distance. Optical fibers pass though crossings inside a labyrinth path designed for radiation safety. For the sake of data accuracy, all the connecting fibers, albeit over a relatively long route, needed shielding with lead bricks to avoid degradation and optical loss. The optical sensors to be analyzed were placed in front of the water pool storing the gamma source and needed proper temperature insulation. Temperature was carefully monitored with 20 m long thermocouples inside and outside the insulating box containing the gratings, which were fixed on plastic frames to avoid strain-induced artifacts. One of the limitations of this setup is the necessity to periodically retract the 60-Co rods in the water tank in order to unload and reload products to be irradiated. Therefore, the exposure of the sensors is not continuous and partial recoveries were observed during irradiation, with a consequent effect on the reproducibility of the results with regard to the accumulated dose. Temperature and humidity fluctuations were recorded and corrections needed to be applied to the data as required for each experiment. Dose rate stability had to be carefully analyzed because of the stop sequences. The dose rate, depending on the position of the setup with respect to the source and facility geometry, was around ten times lower than the dose rate of the irradiation setup described below. The meant the experiments for the same accumulated doses lasted longer and more resources were consumed. Because of the source type, irradiation time, and complicated optical paths, the chance of data errors or experiment failures was significant.

**Figure 2:**
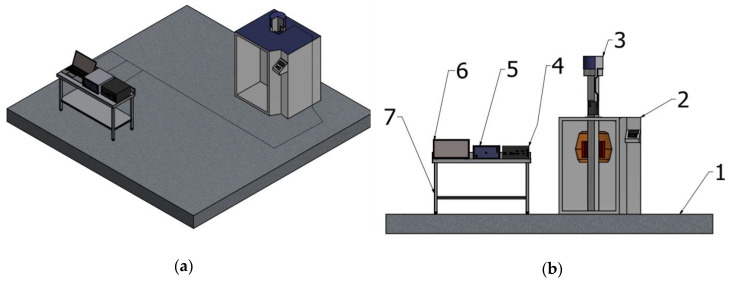
Schematic of the irradiation and measurement setup based on the GC-5000: (**a**) Overall view; (**b**) Detailed view; 1. Ground, 2. GC-5000, 3. Irradiation tube, 4. Optical interrogator, 5. OBR, 6. Laptop, 7. Table support.

**Figure 3:**
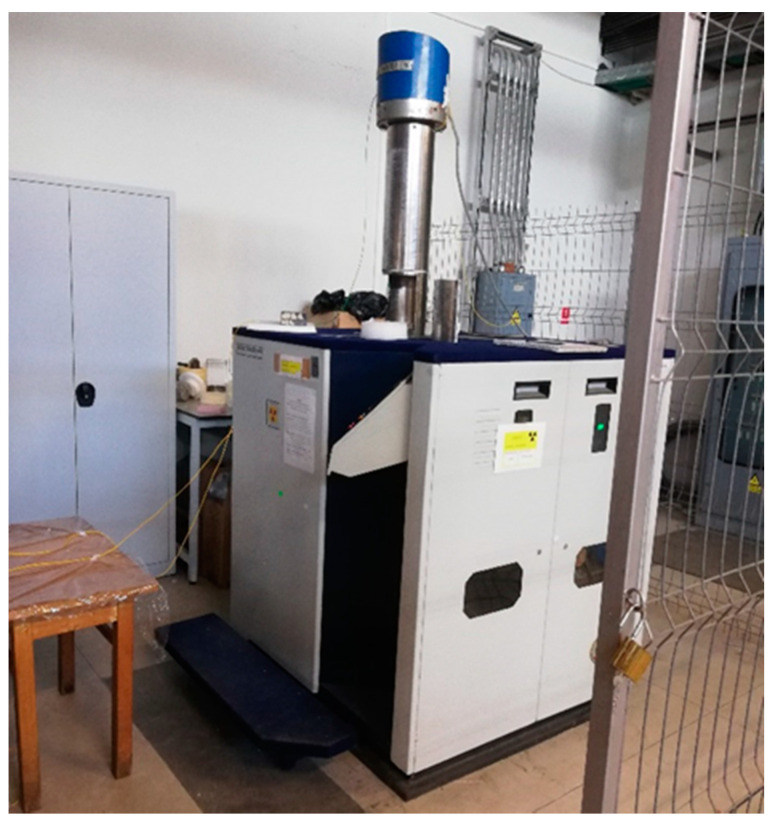
Photo of the irradiation and measurement setup based on the GC-5000.

A new test configuration was designed to avoid the limitations discussed above, and this is illustrated in Figure 2a. The goal is to provide optical fiber irradiation safely, in a customized fashion, to create higher reproducibility and to remotely collect data in real-time. Two single mode fibers were utilized for connections in order to increase the versatility of the setup. Before and after the irradiation, fiber optical transmission was checked via an optical backscatter reflectometer OBR 4600 (LUNA, Roanoke, VA, USA), working in the wavelength range of 1525–1610 nm. Given the sheltered position of the connecting fibers inside the irradiator, no changes in terms of RIA were noticed. The experimental setup is divided into two zones and a detailed view of the components is presented in Figure 2b. The first zone is the operator area, where the interrogation equipment is placed. For this case study, the SM125 optical interrogator (Micron Optics, Atlanta, GA, USA), was utilized for fiber sensor acquisition. The unit has four channels for the simultaneous measurement of different sensors, operating in the wavelength range of 1510–1590 nm. The fiber device is connected to a single channel, where a tunable light source provides the illumination whereas the light reflected by the device is collected by the detector at the same channel. It can detect the resonance wavelength of fiber gratings with a 1 pm resolution and a maximum acquisition rate of 1 s. The second area of the setup includes the GC-5000 irradiator (B.R.I.T., Maharashtra, India) and the optical components under test. The distance between the two is around two meters, which makes it possible to use short connecting fibers between the interrogation units and the devices under testing. A photo of the setup is shown in Figure 3.

**Figure 4:**
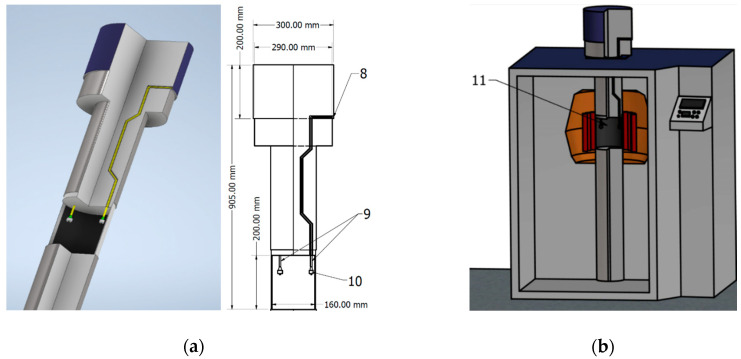
Schematic details of the irradiator tube: (**a**) Section views, 8. Cable Channel, 9. Connection fibers, 10. Fiber connectors; (**b**) Exposure zone of gamma chamber, 11. Area surrounded by Co-60 bars.

The optical fiber devices to be tested are placed inside a 5000 cm^3^ space at the bottom of the tube (3) shown in Figure 2b, which is completely accessible during the preparation of the experiment. The components are placed in the middle with respect to the cylindrically arranged gamma source, so that the radiation is homogenous. In order for the connecting fibers to reach the devices under test, they were inserted through two channels from the top of the tube and exited in the space below via passage holes. Because the diameter of the cable passage channel is too small for an FC/APC connector to fit, the fibers were spliced to the connectors after passing the channels, with the splice section being pulled outside of the chamber for safety reasons. The passage through the irradiator tube can be clearly seen in Figure 4a: the passage aperture has two angled chamfers to prevent irradiation outside the dedicated zone. Special care was taken to verify the absence of bend-induced optical power losses. The irradiator operating principle implies the tube (Figure 4a) is automatically lowered into the irradiation area from a control panel, after the experimental parameters have been set. Figure 4b schematically shows the irradiator tube set in the exposure zone in the middle of the cylindrically arranged Co-60 bars, thus obtaining optimum radiation homogeneity. The chamber hosting the components remains in the current position for the programmed time, thus exposing the devices under test.

Although the GC-5000 implies simpler procedures for research experiments, radiation safety standards were applied in both scenarios reported here according to the International Atomic Energy Agency (IAEA), and only qualified and certified staff could operate the irradiators. The interrogation equipment can be placed 1–2 m away from the chamber as compared to more than 20 m in the case of the industrial irradiator. During both the setup placement and the irradiation, it is not possible for the operator to have any contact with the exposure zone due to the compactness of the irradiator. Moreover, irradiation time is set remotely and the irradiation stops automatically. The samples can be safely removed when the irradiator tube shown in Figure 4a, reaches outside the chamber, leaving no risks of supra-exposure.

### 2.2. Fabrication of Long Period Fiber Gratings

In the present case study, optical fiber long period gratings were selected to evaluate the effectiveness of the irradiation setup. LPG sensors are fabricated by inducing a periodic perturbation in the refractive index and/or geometry of an optical fiber. The period of the perturbation Λ is between 100 and 1000 µm and promotes the power coupling between the core mode and co-propagating cladding modes. As a result, the transmission spectrum of the fiber presents a series of attenuation bands located at the wavelengths that satisfy the phase-matching condition [37]. The properties of these rejection bands are dependent on fiber parameters, grating properties, as well es external conditions of temperature, strain, and the surrounding medium refractive index [38]. Hence, by monitoring the spectral position and/or depth of the attenuation bands, these devices have been applied in a wide range of sensing applications [39,40].

In this work, LPGs were fabricated by the electric arc discharge method at “Parthenope” University of Naples, as detailed elsewhere [41]. Briefly, EAD is a simple and flexible technique that enables the fabrication of LPGs in standard and unconventional fibers, where the periodic modulation of the fiber properties is achieved by localized temperature increases due to the electrical discharge occurring between two electrodes. The discharge is periodically repeated and it is combined with a mechanical action, such as like pulling the fiber by constant axial tension, to ensure a repeatable process [41]. 

**Figure 5:**
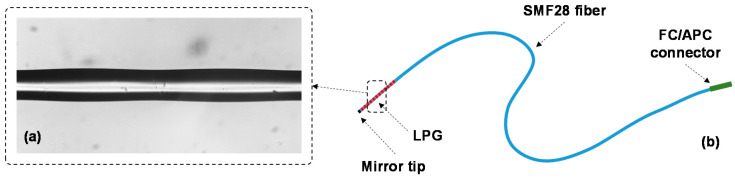
(**a**) Microscope image of the LPG fiber region showing period tapering due to EAD treatment; (**b**) Schematic of LPG fiber sensor configuration tested in the GC-5000 setup.

Here, the LPGs were inscribed in standard SMF28 fiber (with D_co_ = 8.2 µm, D_cl_ = 125 µm, MFD = 10.4 ± 0.8 µm @ 1550 nm, and NA = 0.14) provided by Oz Optics Ltd., Canada, with a period Λ = 628 µm, in order to have a deep attenuation band corresponding to the 3rd order cladding mode located around 1560 nm, where following analyses will be focused. Figure 5a presents a microscope image of the fiber with LPG tested in the GC-5000 setup, and it shows the periodic tapering of the fiber due to the EAD effect. Subsequently, it was modified to operate in a single-ended configuration [42], which is schematically plotted in Figure 5b. During the test, the grating spectra were collected by a connected SM125 interrogator with a repetition rate of 30 s.

## 3. Results

**Figure 6:**
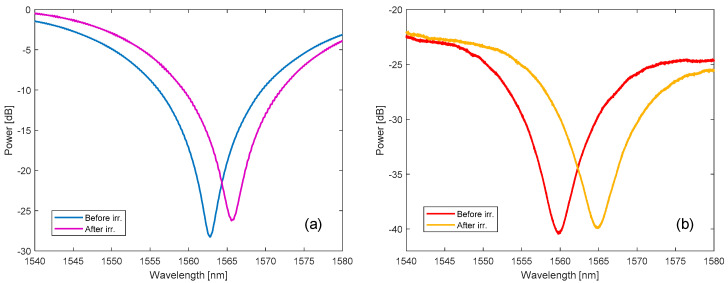
Comparison of the spectra of the LPGs before and after the irradiation performed using the: (**a**) Industrial irradiator SVST Co-60; (**b**) Novel proposed setup based on GC-5000.

The first analysis regards the off-line measurement of LPG power spectra acquired before and after irradiation, to evaluate the radiation induced changes. The results are reported in Figure 6, and the industrial SVST Co-60 irradiator (Figure 6a) is compared to the currently proposed setup based on the customized GC-5000 (Figure 6b). As can be observed, in both cases the LPG resonance wavelength associated to the 3rd order cladding mode shifted to higher wavelengths as a consequence of an increase in the core refractive index due to the radiation, which is in agreement with the literature [23]. The shift was equal to 3.7 nm after a total dose of 35 kGy in the case of the SVST Co-60 irradiator, whereas for the GC-5000 setup it was 4.7 nm after a total dose of 52.6 kGy. 

We recently developed a method that combines these experimental data with numerical simulations to estimate the radiation induced changes in the fiber [22]. Hence, we found refractive index variations in the core of around 1.5 × 10^−5^ and 1.9 × 10^−5^ in the first and second case, respectively. Moreover, the temperature sensitivity of the gratings was compared before and after the irradiation by arranging the grating in a controlled temperature chamber [41]: for the grating in Figure 6a it increased from 50.5 to 53.8 pm/°C, whereas it increased from 52.9 to 62.5 pm/°C in the case of the LPG depicted in Figure 6b. As expected, the off-line spectral results obtained with the two different setups are very similar; any slight changes can be attributed to the different dose rates.

**Figure 7:**
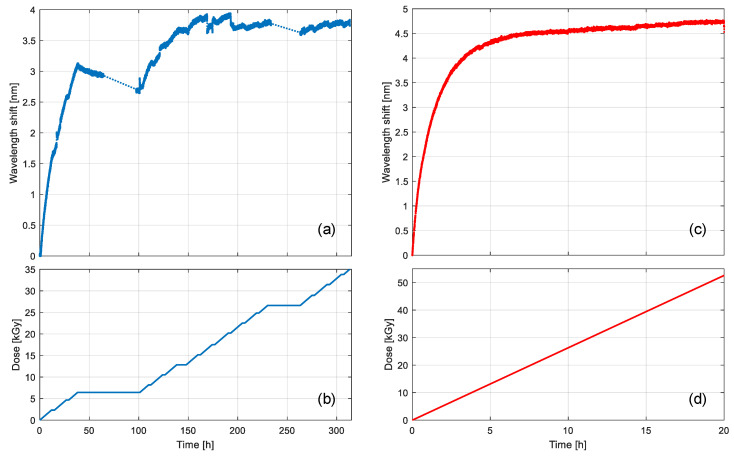
Real-time measurement of the LPGs during gamma irradiation: (**a**) wavelength shift and (**b**) irradiation profile using the industrial SVST Co-60 irradiator; (**c**) wavelength shift and (**d**) irradiation profile using the GC-5000 custom setup.

To highlight the advantages of the setup, we examined the real-time measurement of the LPG wavelength shift during the irradiation. Figure 7a illustrates the variations in the resonance wavelength measured during irradiation in the industrial irradiator. The test was performed at a dose rate of 0.18 kGy/h up to a total dose of 35 kGy, at around 23 °C temperature, and it lasted around 300 h, as seen in the irradiation profile (i.e., dose versus time) reported in Figure 7b. The grating attenuation band shifted to higher wavelengths, in agreement with the literature [23]: the response showed an approximate linear trend at lower doses, subsequently the slope decreased until it reached a saturation level at about 15 kGy. Several breaks in the irradiation can be observed in Figure 7b, which resulted in a partial recovery of the wavelength shift in Figure 7a due to bleaching mechanisms [32], as the source had to be periodically retracted in the water tank. It is also worth noting that total irradiation time is more than 300 h long (i.e., almost two weeks), with obvious impacts in terms of worktime and costs.

Figure 7c reports the resonance shift in the LPG measured during irradiation with the current setup. The results were compensated by the temperature effects, which were precisely measured in real-time using the chamber built-in system, without the need for external reference sensors. The irradiation was performed at a dose rate of 2.63 kGy/h up to a total dose of 52.6 kGy, at around 28 °C, and it lasted 20 h, as shown in Figure 7d. The grating response in Figure 7c is qualitatively similar to that in Figure 7a, but the shape is much smoother in the case of new setup—there are no breaks in the irradiation in Figure 7d and the dose rate is uniform throughout the whole irradiation process, which means no recovery effects were observed, as can be seen in Figure 7c. Also, in this case, the saturation dose is around 15 kGy. The absence of breaks in the irradiation suggests an increase in the reproducibility of the results in terms of dose, moreover, the process is also optimized in terms of time and resources as the irradiation is only 20 h long with a total dose higher than 50 kGy.

**Figure 8:**
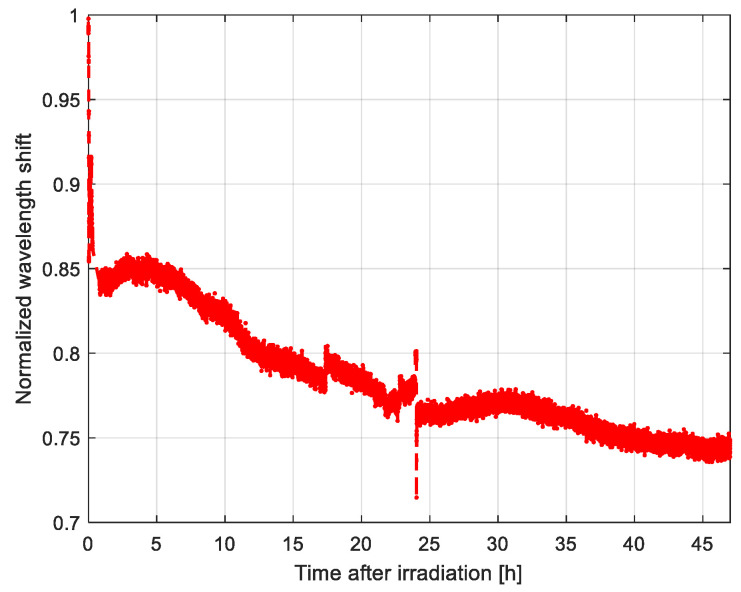
Recovery of the wavelength shift after the irradiation measured using the GC-5000 setup. The wavelength shift is divided by the value at the end of the irradiation (normalized), whereas the time axis is reported starting at the same instant.

Finally, the GC-5000 proposed setup was also used to observe the recovery phase at room temperature after the irradiation, as reported in Figure 8, where part of the radiation induced effects fades due to bleaching mechanisms [23]. Here, the wavelength shift is normalized to the value measured at the end of the irradiation and the time is reported as starting at the same instant. The recovery phase was observed for a long time, i.e., almost 2 days, where the wavelength shift decreased to roughly 75% of the final value, which corresponded to about 3.5 nm. A residual refractive index change after recovery of 1.4 × 10^−5^ was estimated for this wavelength shift by means of our approach based on numerical simulations [22].

## 4. Discussion and Conclusion

In this work, we have reported on the real-time investigation of long period gratings inscribed in standard SMF28 while subjected to gamma-rays using two experimental irradiation configurations. For the first time, to the best of our knowledge, a customized GC-5000 Co-60 (B.R.I.T., India) research gamma irradiator was employed for on-line testing of such optical components up to a dose of 52.6 kGy. This was compared to an industrial SVST Co-60/B gamma irradiator that was utilized for the irradiation of a matching type of LPG with the same characteristics up to 35 kGy. The GC-5000 Co-60, which was modified to allow the investigation of the gratings in real-time, proved to be highly efficiency and demonstrated that it would be more suitable for customized irradiation testing. Its major advantages are summarized as follows:

- The total accumulated dose can be set as to be perfectly suitable for any custom experiment, moreover the irradiation is continuous, providing a very stable dose-rate. Specifically, the dose rate is more than 10 times higher than the industrial SVST Co-60 irradiator (2.63 kGy/h versus 0.18 kGy/h), and thus it proved to be much more efficient in terms of time and resources.
- Given the position of the LPG under testing, that is, in the middle of the 60-Co rods and in a narrow space, the radiation homogeneity is optimal.
- There are no uncontrolled temperature and humidity variations to affect the sensors, hence the temperature profile during irradiation is very well known, measured continuously and similar in all tests.
- No external factors affect the experiments, as compared to industrial facilities where the dose rate is dependent on the products being processed and can vary by 20% depending on the product density and loading pattern in the irradiation containers.
- Post-irradiation monitoring is possible without changing the peripherals and the gratings are kept in the same conditions as during the irradiation.
- The costs of an irradiation session with the GC-5000 based setup are around half k€, whereas they can go up to a few tens k€ for the industrial SVST Co-60/B irradiator.
- Although the same radiation standards were applied in both scenarios, according to the IAEA, the GC-5000 involves simpler procedures with regard to labor protection measures, mainly due to the compactness and full automation of the irradiator.

All of these advantages demonstrate the suitability of the presented setup for research activities involving radiation effects on different types of optical fibers and optical fiber sensors as it provides perfect parameter control, high accuracy and reproducibility of customized metrological experiments in the field.
